# Functionalized Carbon Nanostructures Versus Drug Resistance: Promising Scenarios in Cancer Treatment

**DOI:** 10.3390/molecules25092102

**Published:** 2020-04-30

**Authors:** Manuela Curcio, Annafranca Farfalla, Federica Saletta, Emanuele Valli, Elvira Pantuso, Fiore Pasquale Nicoletta, Francesca Iemma, Orazio Vittorio, Giuseppe Cirillo

**Affiliations:** 1 Department of Pharmacy, Health and Nutritional Sciences, University of Calabria, 87036 Rende (CS), Italy; manuela.curcio@unical.it (M.C.); annafranca.farfalla@gmail.com (A.F.); elvirapnt.ep@gmail.com (E.P.); fiore.nicoletta@unical.it (F.P.N.); francesca.iemma@unical.it (F.I.); 2 Lowy Cancer Research Centre, Children’s Cancer Institute, UNSW Sydney, NSW 2031, Australia; FSaletta@ccia.org.au (F.S.); EValli@ccia.org.au (E.V.); 3 School of Women’s and Children’s Health, Faculty of Medicine, UNSW Sydney, NSW 2052, Australia; 4 ARC Centre of Excellence for Convergent BioNano Science and Technology, Australian Centre for NanoMedicine, UNSW Sydney, NSW 2052, Australia

**Keywords:** carbon nanostructures, carbon nanohybrids, cancer therapy, multi-drug resistance

## Abstract

Carbon nanostructures (CN) are emerging valuable materials for the assembly of highly engineered multifunctional nanovehicles for cancer therapy, in particular for counteracting the insurgence of multi-drug resistance (MDR). In this regard, carbon nanotubes (CNT), graphene oxide (GO), and fullerenes (F) have been proposed as promising materials due to their superior physical, chemical, and biological features. The possibility to easily modify their surface, conferring tailored properties, allows different CN derivatives to be synthesized. Although many studies have explored this topic, a comprehensive review evaluating the beneficial use of functionalized CNT vs G or F is still missing. Within this paper, the most relevant examples of CN-based nanosystems proposed for MDR reversal are reviewed, taking into consideration the functionalization routes, as well as the biological mechanisms involved and the possible toxicity concerns. The main aim is to understand which functional CN represents the most promising strategy to be further investigated for overcoming MDR in cancer.

## 1. Introduction

The USA National Cancer Institute defines cancer as several diseases characterized by an uncontrollable proliferation of abnormal cells invading surrounding tissues [1], with over 10 million new cases diagnosed each year with a survival rate of around 40% [2]. The high incidence of unfavorable prognoses is related to a multitude of factors, including late stage diagnosis, cancer cell plasticity, lack of therapeutic approaches for eradicating disseminated cancer cells, and development of multi-drug resistance (MDR) [3]. On the basis of the underlying developing mechanism, MDR can be classified as intrinsic and extrinsic [4], if depends on acquired genetic alterations in tumor cells [5] or to prolonged exposure to chemotherapy [6], respectively.

The main extrinsic MDR mechanisms involve the reduction of either intracellular drug concentration or activity, and the alteration of cellular apoptotic pathways [7]. More in details, MDR is characterized by the variation of either cell membranes (protein and lipid composition) or cytoplasm (intracellular endocytic vesicles) and nuclei (genetic machinery) features [8].

**Figure 1:**
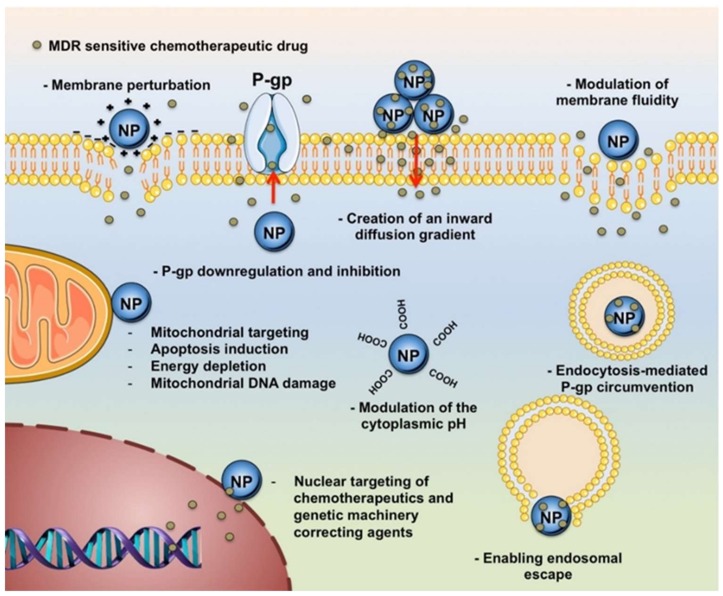
General representation of the main MDR reversal mechanisms by nanoparticle systems (NP). Reproduced with permission from [4]. Copyright Elsevier (2017)

Overcoming MDR is one of the big challenges to ensure the success of chemotherapy, with nanotechnology offering powerful tools for addressing this issue. Different nanomaterials, including metal-based (e.g., iron, silver, and gold nanoparticles), carbon-based (e.g., carbon nanotubes, graphene), and polymer-based (e.g., polymer therapeutics and polymer nanoparticles) materials, have been proposed for various aspects of cancer therapy. Such materials, often combined in composite systems, have gained intense research interest due to their ability to enhance the therapeutic effectiveness and reduce the systemic side effects of conventional cytotoxic drugs [9] (Figure 1).

Carbon nanostructures (CN) consist of sp^2^ carbon atoms with different spatial arrangements, mainly consisting in fullerenes (F, 0D) [10], carbon nanotubes (CNT, 1D) [11], graphene (G, 2D) [12], and graphite/diamond (3D) [13].

G, a flat honeycomb lattice composed of a single layer of hexagonal carbon atoms held together by a backbone of overlapping sp^2^ hybrid bonds, can be assumed as the basic building block for other CN [14].

F are irregular stacked graphene sheets arranged in hollow spherical or ellipsoid structures, where carbon atoms form hexagonal or pentagonal rings. Various forms of fullerenes have been found, including C_60_, C_70_, C_76_, C_80_, C_84_, with sizes ranging from 30 up to 3000 carbon atoms [15]. The most stable fullerene is C_60_, which is composed of 12 pentagonal and 20 hexagonal carbon atom rings.

CNTs can be imaginatively produced by rolling up one single-walled (SWCNT) or multi-walled CNTs (MWCNT) layer of graphene sheet to form cylindrical tubes with a pore diameter <100 nm and a length on the micron scale [16], being closed at the ends with fullerenes halfspheres [17].

Other peculiar classes of CN include quasi spherical graphene structures (graphene quantum dots—GQD) [18], elongated strips of graphene (carbon nanoribbons) [19], rolled graphene sheets with a closed horn-shaped tip (carbon nanohorns) [20], conical cap curved by pentagonal carbon rings (carbon nanocones) [21].

Here, we are giving an overview of the most relevant vehicles based on the CN mainly proposed for the triggered delivery of chemotherapeutics to resistant cancer cells, taking into consideration the biological performances as well as the technological features of the delivery systems. We aim to show how this recent field of research, has contributed to improve the knowledge for developing effective cancer treatments, and what are the potential directions where researchers can focus their future studies to accelerate the translation of their discoveries to clinical trials. By highlighting the strength and the weakness of each CN and derivatization strategy, we aim to show the scientific evidences supporting the hypothesis that the use of G instead of CNT derivatives can address the toxicity concerns and open new perspectives for improving drug efficiency in fighting cancer.

## 2. Carbon Nanostructures and Cancer: Toxicity Concerns and Needs for Tailored Functionalization

CN have aroused great interest among the scientific community for a plethora of applications [22], in physics, chemistry, material science, engineering, electronics, biology, and medicine [23,24] by virtue of their peculiar properties, such as high stability, electrical conductivity, versatile derivatization routes, NIR absorption, and ability to easily penetrate cell membranes. In the biomedical field, CN (CNT, G and C_60_ in particular) represent valuable tools for bio-sensing and bio-imaging [25], as well as for the design of tailored drug carrier systems [26,27]. Their high affinity for organic molecules (independent of molecular weight) confers high drug loading ability, allowing drug stabilization, and improved pharmacokinetic profiles, resulting in enhanced cell uptake [28]. Moreover, when combined with proper materials, CN can modulate the release of a therapeutic agent upon either the application of external stimuli such as magnetic and electric [29,30] fields, or the variation of environmental parameters such pH [31] and temperature [32]. Thus, CN are widely exploited as platforms for delivering bioactive molecules and genes with improved efficiency.

On the other hands, a preliminary functionalization of CN is required, since pristine materials suffer from severe toxic effects due to the lack of solubility in physiological environment. The toxicity of CN mainly arises from the interference with the cell membrane integrity and function, the damage of DNA and RNA, as well as the induction of oxidative stress, inflammatory response, apoptosis, autophagy, and necrosis [33]. In detail, the different CN show specific toxicity profiles, which are relative to their peculiar morphological and shape features, which are affecting cell response to drugs and thus the potential undesired side-effects.

According to the World Health Organisation (WHO), the CNT toxicity concerns come from their fiber-like structure and similarity to asbestos fibers, with carcinogenic effects mainly related to their size [33]. Research data have demonstrated that intra-abdominal injection of long MWCNT determines chronic inflammation of the abdominal wall with the formation of mesothelioma [34]. On the contrary, no inflammation was detected in the case of short MWCNT due to the complete phagocytosis by macrophages, although activated phagocytes can result in the generation of Reactive Oxygen Species (ROS) and thus in late DNA damage [35]. Minimal genotoxicity was recorded upon exposure to low F doses as a consequence of the photosensitizing effect induced by ROS generation, while inflammation was detected at high dosages due to nitric oxide synthase-dependent induction of cyclooxygenase-2 [33]. The high rigidity of pristine G may induce incomplete phagocytosis and thus inflammation and ROS generation, although it is not totally clarified if ROS originate from the its surface or is formed by cellular reactions involving mitochondria and leukocyte [36].

Different approaches have been proposed to improve the water affinity and thus the biocompatibility of CN via the modification of their surface properties [37,38]. The functionalization routes can be divided in two main categories: (i) the covalent attachment of chemical functionalities with change (from sp^2^ to sp^3^) in the hybridization of carbon atoms in the site of reaction [39], and (ii) the noncovalent adsorption/wrapping (π-π stacking) of tailored functional molecules via hydrophobic, electrostatic and Van der Waals interactions, without any chemical changes in the electron patterns of the CN surface [40,41,42].

Different strategies for the covalent functionalization of CN, involving the typical reactivity of the sp^2^ carbon atoms on their surface, are summarized in Table 1.

**Figure 2:**
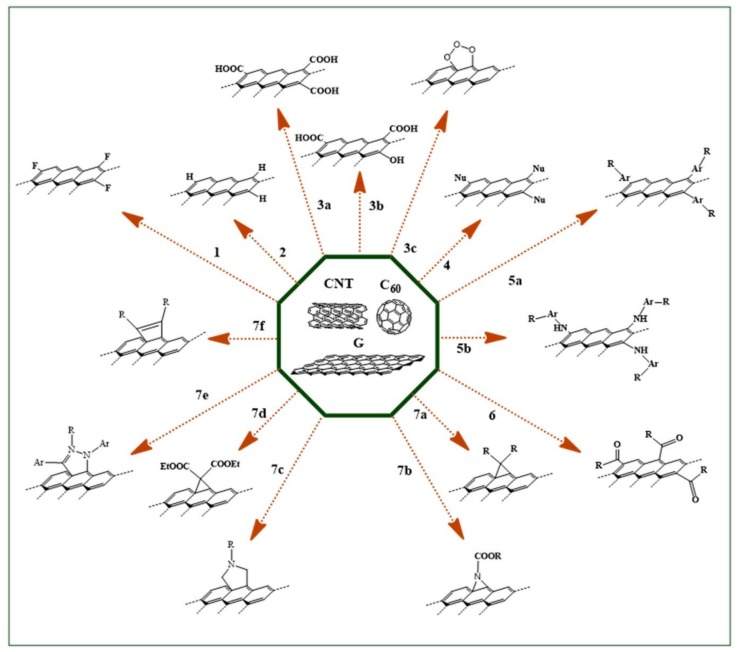
Schematic representation of the main carbon nanostructures covalent functionalization routes. CNT: carbon nanotubes; G: graphene; F: fullerenes. For each reaction, number codes and derivatizing agents are reported in Table 1.

Each approach shows peculiar features in terms of chemical compatibility, reaction conditions, and derivatization degree [43,44], which can be finely tuned according to the specific application needs (Figure 2).

In all cases, intermediate species with high chemical versatility are obtained, with further derivatization processes allowing the fabrication of functional materials for cancer therapy [90]. CNT-based nanosystems proposed for the effective treatment of drug-resistant cancer cells are mainly based on condensation reactions on oxidized CNT (oxCNT), prepared by chemical- [91], photo- [92] or gas- [93] based treatment. Depending on the reaction conditions and the exposure time, such methods are suitable for either the CNT purification from the residual impurities of the synthetic procedures or for the introduction of oxygen-rich functionalities (e.g., hydroxyl and carboxyl groups) on their surface [94] with an increase of water solubility up to 2 mg mL^−1^ [95]. It should be pointed out that, although CNT water dispersions form colloidal suspensions rather than true solutions in the molecular sense, the term “solution” is widely accepted when describing such dispersions [96]. The oxidation reaction starts from carbon bonds at the tips, which are characterized by a higher reactivity due to their larger curvature [97], although the hexagonal cylindrical tube walls are also involved with the formation of defects sites on CNT surface [16]. To avoid the formation of such defects, Prato and co-workers developed a [1,3] cycloaddition reaction of azomethine ylides generated in-situ by thermal condensation of aldehydes and α-amino acids [11]. Under mild reaction conditions, the azomethine ylides are coupled to π-bonds with the formation of pyrrolidine rings, allowing to finely modulate the chemical properties of the side chains on the final product [98]. The advantages of this approach are the simultaneous improvement of the CNT water affinity, the removal of the metal nanoparticles and amorphous carbon impurities, as well as the possibility to thermally remove the introduced organic groups, restoring the original CNT structure [99]. Another key CNT functionalization route is fluorination, defined as the breaking of the conjugated π layers on CNT surface with the formation of C-F bonds. Methods for fluorination mainly consist in the use of fluorine gas mixtures under proper temperature and positive pressure conditions [100] or plasma gas containing fluorine in vacuum [101]. The obtained materials possess a higher solubility in polar solvents (e.g., 1 mg mL^−1^ in alcohol) and are suitable for further derivatization processes by nucleophilic reactions [102].

Similarly to CNT, graphene oxide (GO) is the most used derivative of G. GO is obtained by exfoliation of graphite by treatment with strong oxidizing agents (e.g., Hummers’ method) [103,104]. The exact structure of GO is difficult to determine, but evidences suggest that the sp^2^-hybridized G lattice is interrupted by hydroxyl (−OH), epoxide (−O−), and carbonyl (−C=O) groups, while carboxyl (−COOH) groups are located at the edge [105]. The presence of aromatic network of sp^2^-hybridized carbons and -COOH groups linked to sp^3^-hybridized carbons is responsible for a good hydrophobic to hydrophilic balance of GO derivatives, allowing a high affinity for organic molecules, as well as a high water affinity and thus biocompatibility [106,107]. Furthermore, the oxygen-rich structures act as reactive sites for chemical functionalization with biocompatible and/or bioactive molecules [108].

rGO, a reduced form of GO with lower oxygen content [109], is another valuable G derivative with enhanced ability to load lipophilic species through π-π stacking [110,111]. The availability of different reducing agents and reaction conditions allows the extent of GO reduction and thus the drug-carrier interactions to be finely modulated [112]. Finally, graphene quantum dots (GQDs) are zero-dimensional G derivatives consisting in few layers of G sheets with size less than 20 nm [113]. They are attracting tremendous interest for the preparation of highly engineered carrier systems [114]. GDQ are obtained from large G sheets by chemical oxidation, thermal, ultrasound or oxygen plasma treatments [115].

## 3. Carbon Nanostructures Fighting Multi-Drug Resistance

The employment of nanocarriers, and of those based on CN in particular, have offered different solutions in cancer therapy [116], including the possibility to minimize, circumvent, or even reverse MDR [117] via two main effects:

a) intrinsic MDR reversing properties [118];
b) delivery of MDR reversing agents acting alone or in combination [119].

According to the biological pathways involved in the MDR reversal, the main mechanisms can be summarized as follows:

a) inhibition of drug efflux pumps;
b) increase of intracellular drug concentration and endosomal escape (enhanced uptake);
c) damage of cell membrane and/or intracellular organelles;
d) phototherapy.

In the next sections, we analyze the key examples of CN carrier systems proposed as MDR reversing methods, highlighting both the carrier features (e.g., used CN, preparation method, presence of anchored functional moieties, and/or targeting effect), and the MDR reversion route. Afterwards, the outcomes of the reviewed studies are summarized, and the efficiency of each system is compared respect to their own side effects.

### 3.1. MDR Reversal by Inhibition of Efflux Pumps

A key mechanism involved in either acquired or intrinsic MDR is the ability of integral membrane transporters to expel xenobiotic substances from living cells, including antibiotics and anti-cancer agents [120]. The P-glycoprotein (P-gp or MDR1), the multidrug resistance-associated protein 1 (MRP1), and the breast cancer resistance protein (BCRP) belong to the superfamily of ATP-binding cassette (ABC) transporters [121]. As the name suggests, these proteins transport a broad range of substrates across biological membranes against concentration gradients by using the energy of ATP hydrolysis [122]. P-gp is physiologically expressed in epithelial cells of the major excretory organs (e.g., liver, kidney, lung) and in the capillary endothelial cells at the blood–tissue interfaces, where plays a key role in preventing the entry and/or facilitating the elimination of drugs and toxins [123]. Moreover, since its overexpression in malignant cells’ membrane is often associated with adverse prognosis, extensive efforts have been made for developing effective P-gp inhibitors able to antagonize its activity. Nanoparticle systems offer opportunities for P-gp inhibition by virtue of either their intrinsic inhibitory effect, or the possibility to co-deliver cytotoxic drugs and P-gp inhibitors [122]. In accordance with Wang et al.’s statement [124], since few experimental works have been designed to clearly determine the molecular mechanism at the basis of CN activity in MDR cancer cells, the molecular pathways involved in MDR reversal are an object of diffuse debate among the scientific community [125]. It can be hypothesized that CN enhance the drug uptake allowing an improved intracellular drug concentration, or that they directly inhibit P-gp activity. Experimental and theoretical methods demonstrated negligible C_60_ efflux by P-gp protein, which was confirmed with the absence of P-gp mediated efflux of a fluorescent substrate model [125]. Nevertheless, Shityakov and Föster [126] demonstrated the high affinity of C_60_ to P-gp, thus hypothesizing C_60_ ability to act as competitive substrate to be expelled from cells. In contrast, SWCNT, although possessing high affinity for the P-gp intracellular domains, cannot be effluxed because of unfavorable thermodinamics [126]. The CN employed for MDR reversal via P-gp modulation are summarized in Table 2.

Two key studies demonstrated the P-gp inhibitory activity of CNT loaded [127] or conjugated [128] with fluorescent probe and anticancer drug, respectively. In the first case, the authors showed that a higher efflux of the substrate was detected because of increased P-gp ATPase activity. On the other hand, the conjugation derivative was found to possess a P-gp inhibitory activity due to both CN and loaded P-gp inhibitor (Quercetin). These contradictory results clearly proved the need of further experiments to determine the mechanism of CN driven MDR reversal.

MDR reversal can also occur due to gene expression down-regulation. Wang et al. [124] showed that pristine MWCNT decreased the expression of proto-oncogene c-Myc, (involved in the regulation of ABC gene expression) without damaging cell membrane or inducing oxidative stress. Similarly, Luo et al. [129] reported the ability of GQDs to interact with P-gp C-rich regions (MRP1, and BCRP) resulting in a significant down-regulation of its expression and a significant reversal of Doxorubicin resistance in MCF-7/ADR breast cancer cells. Furthermore, Miao et al. [130] showed the ability of either pristine or oxidized single walled CNT (oxSWCNT) to inhibit cancer proliferation through reduction of tumor micro-vessel density and suppression of the TGFb1-signalling in osteosarcoma stem cells.

### 3.2. MDR Reversal by Enhanced Cellular Uptake

**Figure 3:**
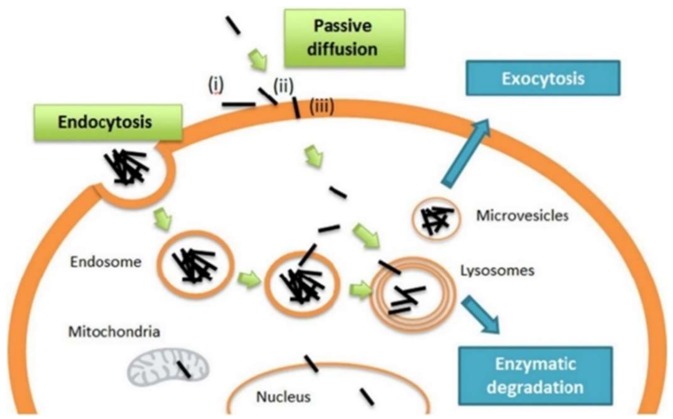
General representation of the main mechanisms involved in the CNT cellular uptake. Reproduced with permission from [137] Elsevier (2016).

The high cell uptake capability of CN can be exploited in the attempt to revert MDR in a Trojian-horse approach where cytotoxic drugs, loaded onto CN, did not directly interact with the membrane machinery thus escaping the efflux transporters [131]. Like nanoparticle systems, CN nanocarriers accumulate in tumor masses through cell junction gaps (around 100–780 nm) of leaky vasculature with poor lymphatic drainage (EPR effect) [132]. Moreover, they are able to cross the cell membrane via dual mechanism involving endocytic pathway or passive diffusion [133]. In detail, individual CNTs are mainly internalized by passive diffusion due to their needle-like shape [134,135], whereas for clustered nanotubes, a clathrin-dependent endocytosis through endosomes followed by trafficking to lysosomes in the perinuclear compartment has been described [136,137] (Figure 3).

Clathrin-dependent mechanism is also accepted as the main mechanism for intracellular internalization of GO and rGO [138]. C_60_ toxic side-effects due to cell membrane penetration have been reported, while endocytosis pathways, typical of hydrophilic C_60_ derivatives uptake, overcome this limitation [139]. The stealth effect can be finely tuned via derivatization of the CN surfaces with a wide range of chemical species, allowing the possibility to spatially control their biological activity by conjugation with targeting elements such as folic acid and antibodies [140,141].

#### 3.2.1. Pristine and Non-Covalently Functionalized CN

Some key examples of MRD reversal by enhanced cellular uptake by pristine and non-covalently coated CN are reported in Table 3.

The ability of pristine SWCNT to penetrate the cell membrane was exploited by Mahmood et al. [142] for the treatment of drug-resistant pancreatic cancer. The authors proved that SWCNT enhanced the cellular uptake of etoposide ETP by 2–5 times when co-administrated. The co-delivery of a cytotoxic agent (doxorubicin—DOX) and a P-gp inhibitor (verapamil—VER) by oxMWCNT, resulted in a significant improvement in the DOX anticancer efficiency due to the increased drug uptake by leukemia drug-resistant cells [143]. The hybridization of CpG oligonucleotide onto pristine SWCNT was used as a strategy to enhance the cell internalization and thus the activation of the innate immune system via Toll-like receptor 9 in a malignant brain cancer model [144]. As a consequence, a selective inhibition of glioma cells migration was observed, while macrophages viability and proliferation remained almost unaltered.

Unmodified GO either enhances the nuclear uptake of cisplatin (CDDP) in several cancer cell lines [145], or delivers oligonucleotides into cells protecting them from enzymatic cleavage [159]. The latter property was exploited by Li et al. [146], who developed a DOX carrier system based on GO modified with two molecular beacons (MBs). The intracellular delivery of MBs silenced the MDR1 and upstreamed erythroblastosis virus E26 oncogene homolog 1 mRNAs. This resulted in an effective inhibition of the P-gp expression and thus in an enhanced efficacy of DOX in resistant breast cancer cells.

The pyrolysis of Fluorinated SWCNT at 1000 °C in an argon atmosphere produced ultra-short single-walled carbon nanotubes (us-SWCNT), which resulted in negligible toxicity when administered in mice [160]. The inner cavity of usSWCNT was filled with CDDP and the resulting device proposed as delivery vehicle for the treatment of breast cancer both in vitro [147] and in vivo [148]. Pluronic 68 (PF 68) was used as coating element to reach an extension of the CDDP release profiles overtime, and an enhancement of drug cytotoxicity against MCF-7 and MDAMB-231 cells was observed in vitro as a consequence of the enhanced cellular uptake [147]. Furthermore, higher CDDP uptake in tumors was detected in in vivo experiments, due to the prolonged blood circulation time facilitating tumor targeting by the EPR effect.

The effect of the CNT diameter on the carrier efficiency against HeLa cells was investigated by Muzi et al. [149]. oxMWCNT with inner diameters of 10 and 38 nm were filled with a hydrophobic Platinum (IV) complex. The authors found that the larger CNT possessed higher cytotoxic properties, whilst the 10nm CNT provided a more prolonged payload release. Interestingly, both carriers were poorly cytotoxic on macrophages and did not induce any pro-inflammatory response.

The correlation between CNT size reduction and enhancement of the anticancer activity was also exploited in the case of GO materials. In detail, GQD were obtained by Fenton reactions of GO and used as nuclear uptake enhancers of CDDP [150,151] and DOX [152] in various solid cancers. pH-dependent vectorization of DOX to the nucleus of drug-resistant breast cancer cells was obtained by DOX loading onto GO nanosheets [153].

GO carriers enter cells via endocytosis and, escaping the drug efflux systems, allow an effective MDR reversal and a significant reduction of MCF-7/ADR viability. This was shown by the high reversal index value, expressed as the ratio between IC_50_ values of free DOX and DOX@GO. Similar results were obtained by a self-assembled G−dextran nanohybrid, fabricated by π−π interactions of GO and hematin-terminated dextran (HDex) [154], or a GO nanocarrier prepared by using hydroxyethyl cellulose (HEC) and polyanionic cellulose (PAC) as nonionic–anionic synergistic surfactants for GO stabilization in serum [155].

Distearoyl-sn-glycero-3-phosphoethanolamine (DISPE)-PEG was used as coating for the construction of SWCNT nanohybrids suitable for the vectorization of paclitaxel (PTX) and ceramide C6 into drug-resistant pancreatic cancer cells [156]. The key study result was the possibility to trigger the intracellular release of the payloads on-demand from the CNT inner core by inductive heating with an external alternating current or pulsed magnetic field. Negligible toxic side effects have been hypothesized due to the retention of drug inside the nanocarriers in the absence of the external stimulation. DISPE-HA coated SWCNT were used for the targeted delivery of epirubicin (EPI) to CD44-overexpressing resistant lung cancer cells [157]. In vitro experiments demonstrated that the system significantly increased the intracellular delivery and retention of EPI through CD44 receptor-mediated endocytosis.

A multifunctional nanocomplex, composed of GO, polyethylenimine (PEI) and poly(sodium 4-styrenesulfonates) (PSS) was used for the combined DOX delivery and miR-21 gene silencing in drug-resistant breast cancer cells [158]. miR-21 over-expression is significantly correlated with drug resistance in breast cancer, thus the simultaneous down-regulation of miR-21 gene and the enhanced cell accumulation of DOX was proposed as a valuable strategy for re-sensitizing resistant cells to the cytotoxic agent.

#### 3.2.2. Covalently Functionalized CN

Kim et al. [161] investigated the effect of different CN conjugation types (e.g., covalent and non-covalent) on DOX localization in cancer cells. For example, DOX was either conjugated to oxMWCNT via amide bond, or absorbed onto PEG wrapped oxMWCNT via π-π stacking. The results showed a lower DOX uptake in normal cells than in cancer cells, while a higher cellular uptake by clathrin-dependent mechanisms was recorded in the case of covalent conjugation. Furthermore, the conjugation was more effective in sustaining DOX release inside cells, while a faster release was detected in the case of absorbed drug, due to the carrier vulnerability in both low acidic (pH < 5.0) and enzymatic environments. A specific intracellular fate was also observed: a non-covalent carrier was more advantageous for mitochondria drug delivery, while a nucleus targeted delivery was obtained with the DOX-conjugated MWCNT. Furthermore, the covalent conjugation resulted in less amount of effluxed DOX from cancer cells, greater apoptosis and cytotoxic activity. Similarly, the pro-apoptotic potential of ginseng secondary metabolites (ginsenoside Rb1 or Rg1) was improved upon conjugation to oxMWCNT [162].

Different covalent functionalization routes involve CN surface modification with polymeric materials and the subsequent loading of the bioactive agents via physical interaction or chemical conjugation to the hybrid carrier (Table 4).

For example, CN covalent functionalization with PEG enhanced the cell internalization of ruthenium polypyridyl complex (RuPOP) [163] and Tumor necrosis factor-related apoptosis-inducing ligand (TRAIL) [164,165] in different cancer cell lines. In the first case, the enhanced cell internalization of PEG-SWCNT resulted in a significant radio-sensitizing effect and in the possibility to kill resistant liver cancer cells by X-ray treatment, while TRAIL efficacy was enhanced by either PEGylated CNT [164] or GO [165]. In the latter case, a pH-responsive co-delivery of DOX to lung and colon cancers was also demonstrated, both in vitro and in vivo [165].

The covalent derivatization of GO with PEG-polyethylenimine (PEI) was proposed for the co-delivery of sorafenib (SOR) and CER for the treatment of drug-resistant liver cancer [166]. PEG-functionalized SWCNT were derivatized via hydrazone linkers with both salinomycin or PTX as anticancer agents and CD44 antibody for the treatment of breast cancer and cancer stem cells (CSC) subpopulation [167]. pH-responsive release mechanism near the acidic tumor microenvironment was observed, and the in vivo therapeutic efficacy was shown in tumor bearing mice, confirming the therapeutic efficacy of the proposed formulation.

By covalent derivatization of oxSWCNT with chitosan-folic acid conjugate (CS-FA), Jia et al. [168] developed a carrier targeting the hypoxic environment of breast cancer. Interestingly, since the adaptation processes developed by cancer cells to proliferate in the hypoxic environment are also at the basis of MDR insurgence [179], oxygen supply by CNT was found to be an effective approach for MDR reversal by counteracting the imbalance between oxygen demand and supply of cancer tissues [180].

The HA ability to target cancer cells was the rationale of Luo’s [169] and Nigam’s [170] research groups, who exploited the fast and high cell internalization rate of GQD for the vectorization of DOX and gemcitabine (GEM) to drug-resistant lung and pancreas cancer cells, respectively. In the first case, PEGylated GQD was proposed as pH-responsive vehicle for the intracellular delivery of DOX, while the conjugation with human serum albumin was found to enhance the tumor infiltration via gp60 pathway for overcoming the inadequate cellular uptake and small half-life of GEM.

rGO nanohybrids obtained by reductive coupling with an enzymatically synthesized dextran-catechin (DEX-CT) conjugate [181] were proposed as pH responsive delivery vehicle of DOX to either drug sensitive (BE(2)C) or drug-resistant (BE(2)C/ADR) neuroblastoma cells [171]. Taking advantage from the ability of CT moieties to down-regulate P-pg expression, authors proved the possibility to synergize the DOX activity in BE(2)C cells and promote resistance reversal in BE(2)C/ADR cells.

Active targeting strategies were proposed by Li et al. [172]. In this study, anti-P-gp SWNTs functionalized with anti-P-gp antibody via amide bonding were used as DOX vehicle for the treatment of leukemia. By comparing the efficiency of the nanocarriers on drug-resistant K562R and drug sensitive K562S cells, a 23-fold higher binding affinity and a specific localization on the cell membrane of K562R cell were recorded, with a 2.4-fold higher cytotoxic activity. Zhang et al. [173] proposed a further upgrade of this concept by co-loading DOX and gambogic acid (GA) as a cytotoxic agent and a P-gp inhibitor, respectively. As a result, high accumulation of anticancer drug in leukemia drug-resistant cells, and a relevant cytotoxic effect due to enhanced apoptosis were recorded both in vitro and in vivo.

A similar approach was used by Nowacki et al. [174], where anti-CD133 antibodies and CDDP complexes were employed as targeting and bioactive agents, respectively. The authors investigated the effect of either physical or chemical loading on peritoneal carcinomatosis.

Zhang et al. [175] reported the covalent functionalization of GO with a gadolinium-labeled polyamidoamine (PAMAM) dendrimer for in vivo imaging and liver cancer targeted therapy based on the synergistic combination of DOX and colchicine (COLC). PAMAM generation 3.0 functionalized GO nanosheets were also proposed for the pH-responsive delivery of DOX in the presence of MMP-9 shRNA in breast cancer cells, reaching a transfection efficiency significantly higher than that obtained with conventional polymeric carriers such as PEI [176]. A similar approach was developed by Cao et al. [177], who employed a FA-chitosan oligosaccharide (CO) conjugated as derivatizing agent. According to this approach, FA acts as targeting unit, while the amino groups of CO are responsible for the delivery of MDR1 siRNA, allowing an effective MDR reversal in breast and lung cancer cells and an improved DOX efficiency. Finally, it should be cited the covalent derivatization of C_60_ through the Bingel reaction as a strategy for the enhanced cellular uptake of CDDP to either drug sensitive or resistant prostate cancer cells [178].

### 3.3. MDR Reversal by Cell Damage

The interaction between nanomaterials and cell environment is related to both their morphological features and surface properties [182].

Fiber-like materials such as CNT where found to possess intrinsic capability to inhibit microtubule dynamics during mitosis, thus reducing the cell replication rate [183]. Furthermore, toxicological studies showed that, upon exposure to CN, an imbalance between the production of reactive oxygen species and their detoxification by biological systems occurs [184]. This phenomenon ignites a cascade of biological responses, including destabilization of cells and mitochondrial membranes and eventually induction of apoptosis [185], mainly through the MAPK and TGF-β signaling pathways [186]. Such intrinsic cytotoxicity can be exploited for contrasting MDR acquisition in cancer cells, due to their fast replication rate and their high sensitivity to oxidative stress [187] (Table 5).

García-Hevia et al. [188] showed that MWCNT can form biosynthetic microtubules with tubulin responsiveness, resulting in severe deficiencies during mitosis, inhibition of cell migrations, and cell death. Interestingly, the same effect was observed in a xenograft model of MDR melanoma, demonstrating the need for further investigations. In another work from the same group [189], 5-Fluoruracil (5-FU) was used as both surface derivatizing element and bioactive agent. The aim was to couple the CNT key features and drug counterpart in a single device, obtaining a synergistic effect between the ability of 5-FU to inhibits cell replication in the “S” phase and the effect of MWCNT on microtubule dynamics.

Lin et al. [190] functionalized GO by chemical oxidation in the presence of NH_3_ and H_2_O_2_ (N-GOs), obtaining a nanomaterial with peroxidase-like activity, able to disproportionate H_2_O_2_ into hydroxyl radicals in the acidic microenvironment of tumor cells, triggering cell necrosis in vitro and in vivo.

A different approach for the modulation of ROS levels in drug-resistant cancer cells involves the functionalization of GO with metal nanoparticles such as Ag [191] and FePt [192] MNPs. In the first case, the authors synthesized rGO-Ag nanosystem using lycopene as reducing agent, and provided scientific evidence that rGO-Ag promotes ROS generation sensitizing human ovarian cancer cells to trichostatinA (TSA) and inducing cell death. In the second study, Fe-Pt was adsorbed onto PEGylated GO to obtain ROS overproduction and synergize metronidazole (MI) and 5-FU toxicity for the treatment of lung cancer cells.

### 3.4. MDR Reversal by Phototherapy

Therapies relying on light are emerging as valuable tools for fighting cancer, by taking advantage of the site-specificity, the poor insurgence of side effects, the absence of cell resistance, as well as the possibility to trigger the release of cytotoxic drugs to the disease site [193].

**Figure 4:**
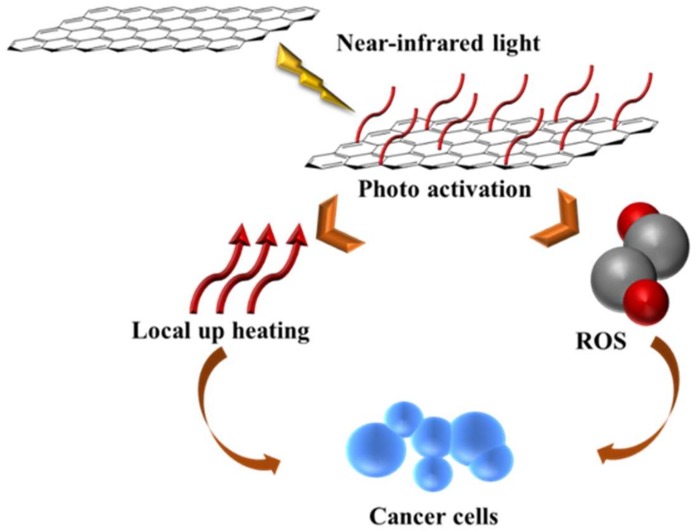
General representation of photothermal therapy by G derivatives. Adapted with permission from [198] copyright Elsevier (2018).

In this regard, phototherapies can be classified in two main categories, photothermal and photodynamic therapy, differing from approach and working mechanism. In the first case, nanomaterials are able to absorb light and produce heat, with the consequent thermal ablation of cancer cells [194]. Photodynamic protocols are based on the generation of singlet oxygen and other ROS, upon exposure to light [195]. In both cases, wavelength in the near-infrared (NIR) region are used since they can reach deeper sites in the body. CNT, C_60_ and GO exhibit high NIR-absorbing capability, and thus are widely proposed as nanocarriers for combined chemo- and photo- therapies (Figure 4) [196,197].

#### 3.4.1. Pristine and Non-Covalently Coated CN

Table 6 summarizes the recent examples of MRD reversal by photo-thermal ablation obtained by the employment of un-modified or non-covalently functionalized CN. C_60_ nanocrystals (nC_60_) are interesting CN obtained upon contact of un-modified C_60_ with water. Under specific conditions, C_60_ forms a water-stable, colloidal aggregate with reported diameters in the 5–500nm range [199]. nC_60_ were found to be suitable materials for the photo-dynamic therapy of thermal ablation of cervix and breast cancer cells [200]. Interestingly, the ROS production and ability to eliminate cancer cells have been shown to significantly increase by wrapping a Neodymium atom in the C_60_ spherical cage [201].

The NIR-absorbing properties of MWNCT in pancreas cancer cells were investigated by Mocan et al. [202], proving that, upon laser treatment, PEGylated MWCNT nanohybrids induced immediate cellular apoptosis as a consequence of increased mitochondrial membrane depolarization.

DISPE-PEG coated CNT were proposed as tools for photo- chemo- therapy protocols involving NIR irradiation of nanohybrid in combination with conventional cytotoxic drugs with the aim to find an effective therapeutic approach for the treatment of chemo- and radio-resistant breast cancer stem cells [203]. An upgrade of this concept was obtained by introducing P-gp antibodies as derivatizing agents for MWCNT surface [204], reaching a targeted thermal ablation in tumor spheroids of MDR cancer cells, with absence of side toxicity on healthy cells. Similarly, P-gp antibodies were conjugated on the surface of oxidized CNH for a combined chemo- and photo-thermal therapy of non-small cell lung cancer [205].

The strategy proposed by Bhirde and co-workers [206] is based on the employment of cholanic acid-derivatized hyaluronic acid (CA-HA) as targeting element in a SWCNT-DOX based therapy for ovarian cancer. Authors stated that the employed noncovalent approach, by preserving the surface integrity and properties of SWCNT, was able to vectorize the drug (due to HA moieties) and improve the cytotoxic drug uptake into either sensitive or resistant cancer cells. A significant reduction of the resistance factor (expressed as the ratio between the IC_50_ values of drug-resistant to drug-sensitive cells) was obtained, and the combination with NIR irradiation was employed for a further enhancement of the therapeutic efficiency in vivo.

Selective vectorization of DOX was achieved by using CS-FA as coating material for oxSWCNT, leading to a NIR-induced intracellular delivery of DOX, with a 12-fold decrease of the IC_50_ of DOX in lung cancer cells [207]. Wang et al. [208] produced anti-CD133 functionalized SWNTs for the selective thermal ablation of glioblastoma stem-like cells in vivo.

The employment of coating with intrinsic biological activity was the rationale of the study by Zhou et al. [209]. By coating SWCNT with glycated chitosan (GCS), the authors developed an immune-adjuvant nanohybrid able to enhance the tumor immunogenicity, leading to remarkable antitumor activity in vivo due to the combined thermal and immunological effects. Nanocarriers, obtained by GO coating with surfactant materials, were developed for the combined photo-thermal treatment of breast and head and neck cancers. PEGylated lipid bilayers [210] and PF 68 [211] were proposed as GO wrapping agents for the synthesis of pH responsive nanocarriers in order to deliver DOX in combination with rapamycin (RAPA) or Irinotecan (IRI), respectively. Upon NIR irradiation, significant apoptosis was induced in breast [210,211] and head and neck [211] cancer cells, resulting in an effective chemo/photo-thermal therapy due to the simultaneous thermal ablation.

#### 3.4.2. Covalently Functionalized CN

Covalent functionalization strategies were also proposed for the development of CN carrier systems with the goal to co-adjuvate cancer therapy by photo-thermal MDR reversal (Table 7).

An improved photo-thermal protocol was developed by Yuan et al. [212]. Here, GO-Gold nanoparticles functionalized with FA and anti- P-gp antibody were used as carrier for MiR-122, allowing an effective induction of apoptosis in drug-resistant HepG2 liver cancer cells, with the possibility to combine drug targeting and controlled release.

The functionalization of GO with HA units conferred targeting ability as well as the possibility to modulate the release of mitoxantrone (MIT) by NIR irradiation [213]. The resulting nanocarrier was also able to act as reversible inhibitor of P-gp, enhancing drug efficiency in either drug-sensitive or drug-resistant breast cancer cells, in vitro and in vivo. A further improvement of PF 68- based GO nanohybrid was proposed by the Wang research group, by developing ROS responsive nanocarriers for the combined vectorization of indocyanine green (ICG) and DOX as photosensitizer and chemo-therapeutic drug, respectively, in breast cancer cells [214,215]. The synthetic approach is based on the formation of a ROS sensitive diselenide bond between GO and a PF68-PAMAM conjugate acting as coating element. Each component contributes to the carrier efficiency: GO enhances the affinity to both photosensitizer and cytotoxic agents, PF 68 confers high stability in physiological environment to the whole system, PAMAM acts as proton sponge allowing lysosomal escape, while the NIR-absorbing properties of both GO and ICG trigger the drug release inside cells by the cleavage of the diselenide bond and eliminate cancer cell by thermal ablation. The covalent modification of GO with PEG in combination with a second polymer, such as poly(allylamine hydrochloride (PAH), resulted in a pH-responsive DOX treatment of resistant breast cancer cells [216].

Zeng et al. [217] proposed the combination of PEG and PEI as GO derivatizing agent to generate a carrier system suitable for the vectorization of DOX and P-gp siRNA to resistant breast cancer cells. The enhanced carrier efficacy was due to the combination of increased loaded DOX, the modulation of P-gp expression by siRNA and the simultaneous thermal ablation upon laser treatment. Zhao et al. [218] used a photosensitizer (2-(1-hexyloxyethyl)-2-devinyl pyropheophorbide-a – HPPH) in combination with PEG for manufacturing hybrid GO nanocarriers for the photo-dynamic therapy of breast cancer in vitro and in vivo. Interestingly, the simultaneous treatment with conventional cytotoxic drugs induced an increase in tumor macrophage infiltration, resulting in an effective cancer eradication.

Alternative strategies for inducing thermal ablation of cancer cells involved the use of radiofrequencies and magnetic fields as activating agents. Sasidharan et al. [219] developed a targeted GO nanosystem by exploiting the overexpression of transferrin (TRF) on drug-resistant cancer cells to trigger thermal ablation of leukemia cells with almost no side-toxicity. Finally, the GO derivatization with superparamagnetic Fe_3_O_4_ NPs carried out for the preparation of DOX nanocarriers able to respond to an external magnetic field, including magnetic hyperthermia, in a combination therapy protocol for drug-resistant breast cancer cells [220].

## 4. Conclusions and Perspectives

MDR represents the main obstacle for the success of chemo-therapeutic protocols in cancer treatment, and great efforts are devoted to the investigation of new strategies for overcoming this phenomenon. Among others, nanoparticle systems such CN, offer solutions by virtue of their ability to interfere with cell structures and functions responsible for intrinsic and acquired MDR. Two main mechanisms are involved, namely the direct modulation of cell pathways and the effective intracellular delivery of MDR reversing agents (e.g., efflux pumps inhibitor or modulators of redox cell state).

Among the different CN, CNT and GO have attracted a higher interest, with promising results in in vitro and in vivo models of different cancers. Furthermore, the availability of several functionalization routes allows the surface properties of CN to be finely modulated by selecting the proper derivatizing agents able to address the specific therapeutic needs.

An overview of the obtained results is given in Table 8, where the outcomes, strength and weakness of each functionalization route is showed in terms of proved success (%) in three categories, namely direct MRD reversal, enhancement of the efficiency of a conventional cytotoxic drug, and the reduction of undesired side effects. Furthermore, for each group, the number of studies (%) covering a specific cancer model and the employed anticancer drug is also reported, in order to give an exhaustive overview of the state of the art in the field.

Although the reviewed studies are very heterogeneous and there is not a biological parameter to be unequivocally used for a direct comparison between the obtained results, some important considerations can be done by considering the score of each item reported in the table.

Most studies investigated the efficiency of carrier systems based on oxCNT, GO, and PEGylated CNT/GO for the treatment of different solid (mainly breast and cervix) and blood cancers. Furthermore, a relevant amount of studies reported the ability of CN materials to directly reverse the MDR allowing, at the same time, the enhancement of the efficiency of a co-administrated drug (mainly DOX) in vitro. These results, being obtained via different molecular mechanisms, can be considered of great interest for scientists working in the field, because of the availability of different strategies, each effecting a peculiar biological pathway. However, despite the high performances recorded in some cases (e.g., carriers based PEGylated CNT or GO functionalized with dendrimers), several issues need to be overcome before hypothesizing a translation of CN nanosystems into clinical practice. At first, more extensive in vivo studies need to clarify the real extent of the obtained results, as highlighted by the differences between in vitro and in vivo success score in the direct MDR reversal and enhanced drug efficiency items of Table 8. Subsequently, the concerns about long-term toxicity must be considered, especially in the case of CNT-based devices, to address the great debate about the benefit of CN in the clinic. Also in this case, the in vivo score of reduced toxic side effects item of Table 8 is very low, even if more encouraging results seems to be achieved using GO as core element of multifunctional vehicles, for which a more homogeneous score is recorded between the effectiveness in MDR reversal, enhancement of drug efficiency, and the reduction of toxicology profiles. To this regard, the introduction of GDQ, coupling high biocompatibility and enhanced cell penetrating behavior, is a step forward for the development of even more interesting materials, although only few examples are available in literature.

Overall, more extensive preclinical and clinical studies are required, in a dynamic interdisciplinary exchange of knowledge between chemists, materials scientists, biologists, and oncologists. Only combining different and complementary expertise, we can hope to succeed in facing the challenges of MRD reversal.

## Figures and Tables

**Table 1:** Main CN covalent functionalization routes.

Reaction			Ref		
N.	Type	Derivatizing Agents	CNT	G	C_60_
1	Halogenation	F_2_	[45]	[46]	[47]
2	Hydrogenation	H_2_	[48]	[49]	[50]
3	Oxidation	a) HNO_3_/H_2_SO_4_	[51]	[52]	[53]
		b) H_2_O_2_	[54]	[55]	[56]
		c) O_3_	[57]	[58]	[59]
4	Nucleophilic Addition	Nu^−^	[60]	[61]	[62]
5	Radical Coupling	a) R-Ar-N_2_^+^	[63]	[64]	[65]
		b) R-Ar-NH_2_	[66]	[67]	[68]
6	Electrophilic Addition	RCOX	[69]	[70]	[71]
7	Cycloaddition	a) R_2_C:	[72]	[73]	[74]
		b) N_3_-COOR	[75]	[76]	[77]
		c) R-NHCH_2_COOH/(CH_2_O)_n_	[78]	[79]	[80]
		d) EtOOCCH_2_COOEt	[81]	[82]	[83]
		e) R-C=N-NH-Ar	[84]	[85]	[86]
		f) -C=C(R)-C(R)=C-	[87]	[88]	[89]

**Table 2:** MDR reversal by CN via inhibition of efflux pumps.

Carrier		Delivery Properties			Cancer Model			Ref
CN	Derivatizing Agent	Bioactive Agent	DL	Responsivity	Tissue	In Vitro	In Vivo	
oxMWCNT	PEG-NH_2_ *Condensation*	---	---	---	Cervix	HeLa	---	[127]
					Liver	HepG2		
						HepG2/R		
					Blood	K562		
						K562R		
oxSWCNT	---	N-TAM-TEG *Condensation*		pH	Breast	MDA-MB-231/R	---	[128]
		Q *π-π Stacking*						
MWCNT	TCM *Coating*	---	---	---	Colon	Caco-2	---	[124]
GQD	---	DOX *π-π Stacking*	---	---	Breast	MCF-7	---	[129]
						MCF-7/ADR		
					Liver	SMMC-7721		
					Colon	Caco-2		
					Blood	HL-60		
SWCNT/oxSWCNT/MWCNT	---	---	---	---	Bone	MNNG/HOS	MNNG/HOS	[130]

DL: drug loading % (*w*/*w*); DOX: doxorubicin; GQD: graphene quantum dots; MWCNT: multi-walled carbon nanotubes; oxMWCNT: oxidized MWCNT; PEG: polyethylene glycol; Q: quercetin; SWCNT: single-walled carbon nanotubes; oxSWCNT: oxidized SWCNT; N-TAM: N-desmethyltamoxifen; TCM: tissue culture medium; TEG: tetraethylene glycol.

**Table 3:** MDR reversal by enhanced uptake of pristine and non-covalently functionalized CN.

Carrier		Delivery Properties			Cancer Model			Ref
CN	Derivatizing Agent	Bioactive Agent	DL	Responsivity	Tissue	In Vitro	In Vivo	
SWCNT	TCM *π-π Staking*	ETP*	11-88^§^	---	Pancreas	PANC-1	---	[142]
oxMWCNT	---	VER *π-π Stacking* DOX*π-π Stacking*	149 164	---	Blood	K562/A02/R	---	[143]
SWCNT	---	CpG	---	---	Brain	K-Luc	---	[144]
						GL261		
					Ovary	OVCAR8		
					Cervix	HeLa		
GO	---	CDDP *π-π Staking*	400	---	Ovary	SCOV-3	---	[145]
					Cervix	HeLa		
					Prostate	Tramp-C1		
					Lung	A549		
					Colon	CT26		
GO	ASO *Hybridization*	DOX *π-π Staking*	35.25	---	Breast	MCF-7/ADR	MCF-7/ADR	[146]
usSWCNT	---	CDDP *Filling*	6.4	---	Breast	MCF-7	---	[147]
	PF108 *π-π Staking*					MDA-MB-231		
usSWCNT	---	CDDP *Filling*	6.4	---	Breast	---	MCF-7	[148]
	PF108 *π-π Staking*						MDA-MB-231	
oxMWCNT	---	Pt(IV) *Filling*	37	---	Cervix	HeLa	---	[149]
GQD	---	CDDP *π-π Staking*	0-50	---	Liver	SMMC-7721	---	[150]
					Cervix	HeLa		
					Lung	A549		
					Breast	MCF-7		
					Stomach	MGC-803		
GQD	---	CDDP *Condensation*	---	pH	Os	HSC3	HSC3	[151]
	PEG-NH_2_ *Condensation*					SCC4	---	
						CAL-27	---	
GQD	----	DOX *π-π Staking*	10	pH	Breast	MCF-7	---	[152]
						MCF-7/ADR		
					Stomach	MGC-803		
GO	---	DOX *π-π Staking*	47	pH	Breast	MCF-7	---	[153]
						MCF-7/ADR		
GO	HDex *π-π Staking*	DOX *π-π Staking*	350	pH	Breast	MCF-7/ADR	---	[154]
GO	HEC/PAC *π-π Staking*	DOX *π-π Staking*	49	pH	Ovary	SCOV-3	---	[155]
						SCOV-3/DDP		
SWCNT	DISPE-PEG *π-π Staking*	PTX/C6/QD *π-π Stacking*	14.3	Magnetic	Pancreas	PANC-1	---	[156]
						MIA PaCa-2		
						L3.6		
oxSWCNT	DISPE-HA *π-π Staking*	ERU *π-π Stacking*	45	pH	Lung	A549	---	[157]
						A549/TXR		
GO	PEI/PSS *π-π Staking*	DOX *π-π Staking* Anti-miR-21	---	---	Breast	MCF-7	---	[158]
						MCF-7/ADR		

* Co-administration with CN; § CNT/drug; DL: drug loading %; ASO: anti-sense oligonucleotide; C6: ceramide C6; CDDP: cisplatin; DISPE: distearoyl-sn-glycero-3-phosphoethanolamine; DOX: doxorubicin; ERU: epirubicin; ETP: etoposide; GO: graphene oxide; GQD: graphene quantum dots; HA: hyaluronic acid; HDex: hemathin-dextran; HEC: hydroxyethyl cellulose; MWCNT: multi-walled carbon nanotubes; oxMWCNT: oxidized MWCNT; PAC: polyanionic cellulose; PEG: polyethylene glycol; PF: Pluronic F; PSS: poly(sodium 4-styrenesulfonates); PTX: paclitaxel; QD: quantum dots; SWCNT: single-walled carbon nanotubes; OxSWCNT: oxidized SWCNT; TCM: tissue culture medium; UsSWCNT: ultra-short SWCNT; VER: verapamil.

**Table 4:** MDR reversal by enhanced cellular uptake of covalently functionalized CN.

Carrier		Delivery Properties			Cancer Model			Ref
CN	Derivatizing Agent	Bioactive Agent	DL	Responsivity	Tissue	In Vitro	In Vivo	
oxMWCNT	---	DOX *Condensation*	112	pH	Lung	A549	---	[161]
	PEG *π-π Stacking*	DOX *π-π Stacking*	31.4		Breast	MDA-MB-231		
oxMWCNT	RB1 *Condensation*	---	25	---	Breast	MCF-7	---	[162]
	RG1 *Condensation*	---			Pancreas	PANC-1		
oxMWCNT	PEG-NH_2_ *Condensation*	RuPOP *π-π Stacking*	9.8	pH X-ray	Liver	HepG2	---	[163]
						R-HepG2		
oxSWCNT	PSE- PEG-NH_2_ *Condensation*	TRAIL *Condensation*	61	---	Liver	HepG2	---	[164]
					Colon	HCT116		
					Lung	H1703		
GO	NH_2_-PEG-N_3_ *Condensation*	DOX *π-π Stacking* TRAIL *Condensation*	78 8	pH	Lung	A549	A549	[165]
					Colon	LoVo	---	
GO	H_2_N-PEG-PEI *Condensation*	CER *Ionic* SRB*	---	---	Liver	HepG2	---	[166]
						HuH7	HuH7	
							HuH7-SR	
						HepG2	---	
oxSWCNT	PEG-HBA/PEG-CD44 Ab *Condensation*	PTX *Condensation*	180	pH	Breast	MDA-MB-231	MDA-MB-231	[167]
		SAL *Condensation*	170					
oxSWCNT	CS-FA *Condensation*	O_2_ *Complexation*	---	---	Breast	MDA-MB-231	---	[168]
		5-FU* ERU* PRU* PTX* CBPT*	3.3^§^ 20^§^ 125^§^ 21^§^ 10^§^					
						ZR-75-1		
GQD	HA-PEG-NH_2_ *Condensation*	DOX *π-π Stacking*	30	pH	Lung	A549	---	[169]
GQD	HA-HSA NPs *Condensation*	GEM *π-π Stacking*	16	---	Pancreas	Panc-1	---	[170]
rGO	DEX-CT *Redox coupling*	DOX *π-π Stacking*	20	pH	Neural Crest	BE(2)C	---	[171]
						BE(2)C/ ADR		
oxSWCNT	P-gp Ab *Condensation*	DOX *π-π Stacking*	20	NIR	Blood	K562	---	[172]
						K562R		
oxMWCNT	P-gp Ab *Condensation*	DOX *π-π Stacking* GA *π-π Stacking*	39.4 30.3	pH	Blood	K562/A02/R	K562/A02/R	[173]
oxSWCNT	CD133 Ab *Condensation*	CDDP *π-π Stacking/Condensation*	66	---	Skin	B16-F10	B16-F10	[174]
		Pt(IV) *π-π Stacking/Condensation*	66					
GO	FA-PAMAM-DTPA *Condensation*	DOXn*π-π Stacking* COLCn*π-π Stacking*	154 154	pH	Liver	HepG2	HepG2	[175]
GO	PAMAM *Condensation*	DOX *π-π Stacking* sRNA *Hybridization*	28.6 5	pH	Breast	MCF-7	---	[176]
GO	FA-CO *Condensation*	DOX *π-π Stacking* sRNA *Hybridization*	56	pH	Breast	MCF-7	---	[177]
						MCF-7/ ADR		
					Lung	A549		
C_60_	Br-C- (COOEt)_2_ *Bingel*	CDDP*			Prostate	PC-3	PC-3R	[178]

* Co-administration with CN; § CNT/drug; DL: drug loading % (*w*/*w*); 5-FU: 5-fluorouracil; Ab: antibody; CBPT: carboplatin; CDDP: cisplatin; CER: ceramide; CO: chitosan oligosaccharide; COLC: colchicine; CS: chitosan; CT: catechin; DEX: dextran; DOX: doxorubicin; DTPA: diethylenetriamine pentaacetate; ERU: epirubicin; FA: folic acid; GA: gambogic acid; GEM: gemcitabine; GO: graphene oxide; HA: hyaluronic acid; HBA: 4-hydrazinobenzoic acid; HSA: human serum albumin; MWCNT: multi-walled carbon nanotubes; oxMWCNT: oxidized MWCNT; NPs: nanoparticles; PAMAM: polyamidoamine; PEG: polyethylene glycol; PEI: polyethylenimine; POP: polypyridyl; PRU: pirarubicin; PSE: 1-pyrenebutanoic acid; PTX: paclitaxel; RB1: RB1 ginsenoside; RG1: RG1 ginsenoside; rGO: reduced graphene oxide; SAL: salinomycin; SRB: sorafenib; SWCNT: single-walled carbon nanotubes; oxSWCNT: oxidized SWCNT; TRAIL: tumor necrosis factor-related apoptosis-inducing ligand.

**Table 5:** MDR reversal by CN via cell damage.

Carrier		Delivery Properties			Cancer Model			Ref
CN	Derivatizing Agent	Bioactive Agent	DL	Responsivity	Tissue	In Vitro	In Vivo	
MWCNT	TCM *π-π Stacking*	---	---	---	Skin	B16-F10	B16-F10	[188]
MWCNT	5-FU *π-π Stacking*		3	---	Skin	---	B16-F10	[189]
					Cervix	HeLa	---	
GO	NH_3_ *Oxidation*	---	---	pH	Cervix	HeLa	HeLa	[190]
rGO	Ag NPs *Redox Coupling*	TSA *π-π Stacking*	100	---	Ovary	SKOV-3	---	[191]
GO	PEG *Condensation* FePt MNPs *π-π Stacking*	MI *π-π Stacking* 5-FU *π-π Stacking*	12.3 9.5	O_2_	Lung	A549	---	[192]
						H1975		

DL: drug loading % (*w*/*w*); 5-FU: 5-fluorouracil; GO: graphene oxide; MI: metronidazole; MNPs: magnetic nanoparticles; MWCNT: multi-walled carbon nanotubes; NPs: nanoparticles; PEG: polyethylene glycol; rGO: reduced graphene oxide; TCM: tissue culture medium; TSA: trichostatin A.

**Table 6:** MDR reversal by photo-thermal ablation induced by pristine and non-covalently functionalized CN.

Carrier		Delivery Properties			Cancer Model			Ref
CN	Derivatizing Agent	Bioactive Agent	DL	Responsivity	Tissue	In Vitro	In Vivo	
nC_60_	---	DOX*	---	---	Cervix	HeLa	---	[200]
					Breast	MCF-7/ADR		
nC_60_	Nd *Encapsulation*	---	---	---	Cervix	HeLa	---	[201]
						H1975		
MWCNT	PEG *π-π Stacking*	---	---	NIR	Pancreas	PANC1	---	[202]
oxMWCNT	H_2_NC_2_H_4_NH_2_ *Amidation* DISPE-PEG *π-π Stacking*	PTX*	---	NIR	Breast	HMLER	---	[203]
		SAL*				HMLER CSC	HMLER CSC	
		17DMAG*						
oxSWCNT	DISPE-PEG/P-gp Ab *π-π Stacking*	---	---	---	Fibroblast	3T3-MDR1	---	[204]
					Ovary	NCI/ADR		
oxCNH	PEG/P-gp Ab *π-π Stacking*	ETP *Filling*	---	NIR	Lung	A549		[205]
						A549R	A549R	
SWCNT	CA-HA *π-π Stacking*	DOX *π-π Stacking*	300	---	Ovary	OVCAR8	---	[206]
						OVCAR8/ADR	OVCAR8/ADR	
oxSWCNT	CS-FA *π-π Stacking*	DOX *π-π Stacking*	33.3	NIR	Lung	A549	A549	[207]
SWCNT	CS-CD133 Ab *π-π Stacking*	---	---	NIR	Brain	GMB-CD133^+^	GMB-CD133^+^	[208]
						GMB-CD133^-^	GMB-CD133^-^	
SWCNT	GCS *π-π Stacking*	---	---	NIR	Breast	EMT6	EMT6	[209]
GO	PEGylated Liposome *Encapsulation*	DOX *π-π Stacking* RAPA *π-π Stacking*	10 10	pH	Breast	MCF-7	---	[210]
						MDA-MB-231		
						BT4T4		
GO	PF 68	DOX *π-π Stacking* IRI *π-π Stacking*	7 7	pH	Breast	MCF-7	---	[211]
						MDA-MB-231		
					Head/ Neck	SCC-7		

* Co-administration with CN; DL: drug loading % (*w*/*w*); 17DMAG: 17-(dimethylaminoethylamino)-17-demethoxygeldanamycin; Ab: antibody; nC_60_: C_60_ nanocrystals; CA cholanic acid; CNH: carbon nanohorns; CS: chitosan; DISPE: distearoyl-sn-glycero-3-phosphoethanolamine; DOX: doxorubicin; ETP: etoposide; FA: folic acid; GCS: glycated chitosan; GO: graphene oxide; HA: hyaluronic acid; IRI: irinotecan; MWCNT: multi-walled carbon nanotubes; NIR: near infrared radiation; oxCNH: oxidized CNH; oxMWCNT: oxidized MWCNT; oxSWCNT: oxidized SWCNT; PEG: polyethylene glycol; PF: Pluronic F; PTX: paclitaxel; RAPA: rapamycin; SAL: salinomycin; SWCNT: single-walled carbon nanotubes.

**Table 7:** MDR reversal by photo-thermal ablation induced by covalently functionalized CN.

Carrier		Delivery Properties			Cancer Model			Ref
CN	Derivatizing Agent	Bioactive Agent	DL	Responsivity	Tissue	In Vitro	In Vivo	
GO	P-gp Ab *Condensation* FA-Au NPs *π-π Stacking*	MiR-122 *Hybridization*	---	---	Liver	Hep-G2/ADR	Hep-G2/ADR	[212]
GO	HA *Condensation* PF 68 *π-π Stacking*	MIT *π-π Stacking*	3	pH	Breast	MCF-7	MCF-7	[213]
						MCF-7/ADR	MCF-7/ADR	
GO	PF 68-PAMAM *Diselenide*	ICG *π-π Stacking*	52.1	ROS	Breast	MCF-7	---	[214]
						MCF-7/ADR		
GO	PF 68-PAMAM *Diselenide*	ICG *π-π Stacking*	52.1	ROS	Breast	MCF-7	---	[215]
						MCF-7/ADR	MCF-7/ADR	
GO	PEG-PAH *Condensation*	DOX *π-π Stacking*	50	pH	Breast	MCF-7	---	[216]
						MCF-7/ADR		
GO	FA-PEG-PEI *Condensation*	DOX *π-π Stacking* sRNA *Hybridization*	---	pH	Breast	MCF-7	---	[217]
						MCF-7/ADR		
GO	PEG-NH_2_ *Condensation* HPPH *π-π Stacking*	CTX *π-π Stacking*	1	NIR	Breast	4T1	4T1	[218]
		DOX *π-π Stacking*						
		DTX *π-π Stacking*						
		5-FU *π-π Stacking*						
GO	TRF *Condensation*	---	---	---	Blood	K562	---	[219]
						K562R		
GO	Fe_3_O_4_/MnO_x_ *Redox Coupling*	DOX *π-π Stacking*	38	pH Redox Magnetic	Breast	MDA-MB-231	---	[220]
						MCF-7/ADR		

5-FU: fluorouracil; Ab: antibody; CTX: cyclophosphamide; DOX: doxorubicin; DTX: docetaxel; FA: folic acid; GO: graphene oxide; HA: hyaluronic acid, HPPH: 2-(1-hexyloxyethyl)-2-devinyl pyropheophorbide a; ICG: indocyanine green; MIT: mitoxantrone; NPs: nanoparticles; PAH: poly(allylamine hydrochloride); PAMAM: polyamidoamine; PEG: polyethylene glycol; PEI: polyethylenimine; PF: Pluronic F; TRF: transferrin.

**Table 8:** Outcomes, strength and weakness of CN based system proposed for MDR reversal reviewed in this paper. Percentages are calculated based on the total studies, with some papers covering more studies simultaneously.

CNs	Deriv	Ref	Total Studies	Drug	Cancer Model	Direct MDR Reversal	Enhanced Drug Efficiency	Reduced Side Effects
				Studies (%)		Success (%)		
F	---	[178] [200] [201]	4	None (25) DOX (50) CDDP (25)	Cervix (50) Prostate (25) Breast (25)	75 * 25 ^#^	75 * 25 ^#^	0 * 0 ^#^
CNT	---	[124] [128] [142] [144] [147] [148] [188] [189]	11	None (46) 5-FU (18) CDDP (18) TAM (9) ETP (9)	Breast (28) Cervix (18) Skin (18) Brain (9) Ovary (9) Colon (9) Pancreas (9)	55 * 18 ^#^	36 * 18 ^#^	0 * 9 ^#^
CNT	Ox	[130] [143] [149] [161] [162] [164] [172] [173] [174]	16	None (6) DOX (30) TRAIL (19) RB1 (13) RG1 (13) Pt(IV) (13) CDDP (6)	Breast (18.5) Blood (18.5) Lung (13) Pancreas (13) Skin (13) Cervix (6) Bone (6) Liver (6) Colon (6)	50 * 25 ^#^	94 * 19 ^#^	31 * 0 ^#^
GO	---	[129] [145] [146] [150] [151] [152] [153] [190] [191] [212] [220]	23	None (22) CDDP (48) DOX (26) TSA (4)	Breast (26) Cervix (13) Liver (13) Ovary (9) Lung (9) Stomach (9) Colon (9) Blood (4) Prostate (4) Os (4)	12 * 4 ^#^	18 * 2 ^#^	10 * 1 ^#^
CNT	PEG	[127] [161] [163] [167] [202]	9	None (45) DOX (22) PTX (11) SAL (11) RuPOP (11)	Breast (34) Liver (23) Cervix (11) Blood (11) Pancreas (11) Lung (11)	56 * 0 ^#^	56 * 22 ^#^	33 * 11 ^#^
CNH	PEG	[205]	1	ETP (100)	Lung (100)	100 * 100 ^#^	100 * 100 ^#^	0 * 0 ^#^
GO	PEG	[151] [165] [169] [170] [192] [210] [216] [218]	12	DOX (50) 5-FU (17) CDDP (8) GEM (8) CTX (8) DTX (8)	Breast (50) Lung (25) Os (8) Colon (8) Pancreas (8	75 * 42 ^#^	92 * 42 ^#^	42 * 0 ^#^
CNT	Surf	[147] [148] [156] [157] [203] [204]	9	None (22) CDDP (22) PTX (22) SAL (11) 17DMAG (11) ERU (11)	Breast (56) Fibroblast (11) Ovary (11) Pancreas (11) Lung (11)	78 * 33 ^#^	67 * 44 ^#^	56 * 0 ^#^
GO	Surf	[211] [213]	3	DOX (67) MIT (33)	Breast (67) Head and Neck (33)	33 * 33 ^#^	100 * 33 ^#^	0 * 0 ^#^
GO	PEI	[158] [166] [217]	3	DOX (67) SRB (33)	Breast (67) Liver (33	100 * 33 ^#^	100 * 33 ^#^	0 * 0 ^#^
GO	Dend	[175] [176] [214] [215]	4	DOX (50) ICG (50)	Breast (75) Liver (25)	100 * 50 ^#^	100 * 50 ^#^	25 * 25 ^#^
CNT	PS	[168] [206] [207] [208] [209]	9	None (22) DOX (22) 5-FU (11) ERU (11) PRU (11) PTX (11) CBPT (11)	Breast (67) Brain (11) Ovary (11) Lung (11)	33 * 33 ^#^	78 * 22 ^#^	11 * 0 ^#^
GO	PS	[154] [155] [171] [177]	5	DOX (100)	Breast (40) Ovary (20) Lung (20) Neural Crest (20)	80 * 0 ^#^	100 * 0 ^#^	20 * 0 ^#^
GO	PR	[219]	1	None (100)	Blood (100)	100 * 100 ^#^	0 * 0 ^#^	100 * 0 ^#^

* In vitro; ^#^ In vivo; CN: carbon nanostructure; Deriv: derivatization; 5-FU: 5-fluorouracil; 17DMAG: 17-(dimethylaminoethylamino)-17-demethoxygeldanamycin; CBPT: carboplatin; CDDP: cisplatin; CNT: carbon nanotubes; CTX: cyclophosphamide; DOX: doxorubicin; DTX: docetaxel; ERU: epirubicin; ETP: etoposide; F: fullerenes; GEM: gemcitabine; GO: graphene oxide; ICG: indocyanine green; MIT: mitoxantrone; Ox: oxidation; PEG: polyethylene glycol; POP: polypyridyl; PTX: paclitaxel; PR: protein; PRU: pirarubicin; PS: polysaccharides; RB1: RB1 ginsenoside; RG1: RG1 ginsenoside; SAL: salinomycin; SRB: sorafenib; Surf: surfactant; TAM: tamoxifen; TRAIL: tumor necrosis factor-related apoptosis-inducing ligand; TSA: trichostatinA.
